# Post-COVID-19 Syndrome: The Persistent Symptoms at the Post-viral Stage of the Disease. A Systematic Review of the Current Data

**DOI:** 10.3389/fmed.2021.653516

**Published:** 2021-05-04

**Authors:** Francesca Salamanna, Francesca Veronesi, Lucia Martini, Maria Paola Landini, Milena Fini

**Affiliations:** ^1^IRCCS Istituto Ortopedico Rizzoli, Complex Structure of Surgical Sciences and Technologies, Bologna, Italy; ^2^IRCCS Istituto Ortopedico Rizzoli, Scientific Direction, Bologna, Italy

**Keywords:** COVID-19, long-term symptoms, persistent symptoms, long-term sequalae, virus

## Abstract

Whilst the entire world is battling the second wave of COVID-19, a substantial proportion of patients who have suffered from the condition in the past months are reporting symptoms that last for months after recovery, i. e., long-term COVID-19 symptoms. We aimed to assess the current evidence on the long-term symptoms in COVID-19 patients. We did a systematic review on PubMed, Web of Science, EMBASE, and Google Scholar from database inception to February 15, 2021, for studies on long-term COVID-19 symptoms. We included all type of papers that reported at least one long-term COVID-19 symptom. We screened studies using a standardized data collection form and pooled data from published studies. Cohort cross-sectional, case-report, cases-series, case-control studies, and review were graded using specific quality assessment tools. Of 11,361 publications found following our initial search we assessed 218 full-text articles, of which 145 met all selection criteria. We found that 20.70% of reports on long-term COVID-19 symptoms were on abnormal lung functions, 24.13% on neurologic complaints and olfactory dysfunctions, and 55.17% on specific widespread symptoms, mainly chronic fatigue, and pain. Despite the relatively high heterogeneity of the reviewed studies, our findings highlighted that a noteworthy proportion of patients who have suffered from SARS-CoV-2 infection present a “post-COVID syndrome.” The multifaceted understanding of all aspects of the COVID-19 pandemic, including these long-term symptoms, will allow us to respond to all the global health challenges, thus paving the way to a stronger public health.

## Introduction

As of March 2021, about 117 million people worldwide have been diagnosed with COVID-19, with more than 2.6 million deaths (1). COVID-19 is caused by the novel severe acute respiratory syndrome coronavirus 2 (SARS-CoV-2), a heterogeneous virus that manifests itself with a wide spectrum of symptoms, from asymptomatic to life-threatening and fatal disease (2–7). Interstitial pneumonia is one of the most common features of SARS-CoV-2 and can be complicated by acute respiratory distress syndrome (ARDS), a disease related with high mortality, particularly in elderly people with multiple comorbidities (2, 3). As the pandemic of COVID-19 continues, numerous additional symptoms, such as fever, dry cough, shortness of breath, fatigue, myalgias, nausea/vomiting or diarrhea, headache, weakness, rhinorrhea, anosmia/ageusia, and many laboratory abnormalities, i.e., lymphopenia and elevated inflammatory markers (e.g., erythrocyte sedimentation rate, C-reactive protein, ferritin, tumor necrosis factor-α, IL-1, and IL-6) have been reported (2, 3). Other critical and severe complications of COVID-19 can include impaired function of the heart, brain, lung, liver, kidney, and coagulation system (4–7).

Most of the infected patients completely recovered after COVID-19 infection. However, a substantial proportion of patients who have been infected with SARS-CoV-2 continue to have symptoms long past the time that they recovered from the initial phases of COVID-19 disease. Clinicians worldwide called these long-term effects of COVID-19 “Long-Haul COVID-19” or “Long-term COVID-19” (8–11). In detail, “long-term COVID-19” defines those individuals who have had SARS-CoV-2 infection but do not recover completely over a period of a few weeks (commonly 2–3 weeks) (8–11). Based on the COVID-19 Symptom Study, a study carried-out on more than 4 million people in the US, UK, and Sweden, in which people enter their ongoing symptoms on a smartphone app, around 10% of patients who have tested positive for SARS-CoV-2 virus remain unwell beyond 3 weeks, and a smaller proportion for months (8). Thus, it is becoming clear, that some people who had a SARS-CoV-2 infection, even those described as “mild,” continue to suffer from persisting or cyclical symptoms. However, because COVID-19 is a novel disease, to date, there is not yet consensus on the definition of post-COVID-19 symptoms. Since long-term symptoms and complications have been described for other highly homologous human coronaviruses, i.e., Middle East Respiratory Syndrome (MERS) and Severe Acute Respiratory Syndrome (SARS), to date, it is unknown whether lessons from MERS and SARS are applicable to COVID-19 and the critical question is: “*Do persistent symptoms at the post-viral stage of the disease constitute a post-COVID-19 syndrome (long-term COVID-19) and what are the main persistent symptoms in patients that might cause such a syndrome?*” (12–14). The obvious answer is in research, but to date we do not know what to tell patients when they are asking about the course and prognosis of their ongoing complaints and potential long-term symptoms. Finding a concrete answer to these questions would also provide more information on the COVID-19 disease and enable comprehensive and targeted care to be given to survivors through the development of preventive and effective treatments. Although we are aware that it is too early to completely answer these questions, we believe that some general predictions are now possible, and would help to implement the right public health measures in particular after the pandemic has subsided. Thus, to give a complete overview on the persistent symptoms at the post-viral stage of COVID-19, we carried out a systematic review of the current data considering all types of papers evaluating individual persistent symptoms in mild, moderate, and severe/critical COVID-19 patients. Realizing the long-term sequelae of COVID-19 is imperative for understanding the complete history of disease, truly predicting the growing effect of the disease beyond hospitalization and mortality and defining whether inpatient or post-discharge-specific rehabilitation should be evaluated.

## Methods

### Eligibility Criteria

The PICO model was used to formulate the questions for this study: (1) studies that considered patients with long-term COVID-19 symptoms (Population), (2) studies where the primary aim was to evaluate long-term COVID-19 symptoms in mild, moderate, severe, and critical patients that have a follow-up of at least 14 days (Interventions), (3) studies with or without a control group (Comparisons), (4) studies that reported the long-term COVID-19 symptoms (Outcomes). Studies conducted up to February 15, 2021 were included in this review if they met the PICO criteria.

### Search Strategies

Our systematic review involved a search conducted on February 15, 2021. We performed the review according to PRISMA statement (15). The search was carried out on PubMed, Web of Science, EMBASE, and Google Scholar databases to identify all type of papers on the long-term symptoms of COVID-19. The search was conducted combining the terms COVID-19, persistent symptoms, long-term symptoms, chronic symptoms, enduring symptoms, permanent symptoms. The combination of free-vocabulary and/or MeSH terms for the identification of studies in PubMed, Web of Science, EMBASE, and Google Scholar were reported in Table 1. Reference lists of relevant articles were searched for other potentially appropriate publications.

**Table 1:** Cohort (perspective and retrospective), cross-sectional, case-report, cases-series and case-control studies on long-term lungs symptoms, long-term neurological and olfactory symptoms, and widespread long-term symptoms.

**References**	**Study type**	**Study country and period**	**Patients characteristics (numbers, gender, age)**	**COVID-19 severity**	**Hospitalization**	**ICU admission**	**Baseline COVID-19 symptoms**	**Method of evaluating long-term COVID-19 symptoms**	**Follow-up**	**Long-term COVID-19 symptoms**
***Persistent lungs symptoms and dysfunctions***										
Bellan et al. (18)	Prospective	•Novara, Italy •March 1 and June 29, 2020	•238 patients: •96 females •142 males •Mean age: 61 (50–71)	Mild to-severe	Yes	28 patients	Fever, cough, dyspnea, ageusia, anosmia, diarrhea, arthralgia, myalgia	D_LCO_, score for posttraumatic stress symptoms and for functional impairment	120 days	D_LCO_ reduced to less than 80% of the estimated value in 113 patients and less than 60% in 34 patients. Functional impairmentin 53 patients
Chun et al. (19)	Retrospective	New Haven, CT	•61 patients •44% females 56% males •Mean age: 53 (43–62)	•13 mild •30 non-critical •18 critical	30 patients	18 patients	Dyspnea and cough	Pulmonary function tests, plasma biomarker profiling	45–67 days	Dyspnea (69%), cough (58%). Pulmonary function declined as acute COVID-19 severity increased and not correlate with symptoms. LCN2, MMP-7, HGF were higher in ICU subjects and inversely correlated with pulmonary function
Daher et al. (20)	Prospective	•Aachen, Germany •February–May 2020	•33 patients •11 females •22 males •Mean age: 64 ± 3	Severe	Yes	No	•Increased D-dimer •LDH activity and CRP, ferritin, and IL-6	Body plethysmography, D_LCO_, blood gas analysis (ABG), 6-min walk test (6MWT), echocardiography, laboratory tests	45 days	Reduced D_LCO_ and 6MWT, and persistent fatigue and dyspnea in most patients.
Ding et al. (21)	Retrospective	•Wuhan, China •February–March 2020	•112 patients: •61 females •51 males •Mean age: 55.8	NR	Yes	NR	Fever, dry cough, fatigue, chest distress, dyspnea, myalgia	CT scan	28 days	Abnormalities in 98.1 % of lungs CT scans (ground-glass opacities, crazy-paving pattern, consolidation and linear opacities)
Frija-Masson et al. (22)	Retrospective	•Paris, France •4 March−1 April 2020	•50 patients: •22 females •28 males •Age ≤ 85	•12 mild •17 moderate •16 severe •5 not classified	Yes	8 patients	Respiratory symptoms	Spirometry, functional residual capacity, total lung capacity, D_LCO_ (single breath real-time CO/NH_4_)	30 days	Impaired lung function in 54% of patients (restriction and/or altered D_LCO_), with a mix of restrictive and low diffusion patterns
Hall et al. (23)	Retrospective	•London, UK •May 2020	•200 patients: •38.5 females •61.5% males •Mean age: 54.8 ± 15.0	Moderate to- severe	89 patients	77 patients	NR	Dual energy CT or high-resolution CT, ventilation-perfusion scanning, spirometry, echocardiography and ECG	30–45 days	40% of patients with cardiorespiratory cause of breathlessness, i.e. persistent parenchymal abnormality pulmonary embolism, cardiac complications
Han et al. (24)	Prospective	•Hubei, China •December 25, 2019 - February 20, 2020	•114 patients: •34 females •80 males •Mean age: 54 ± 12	Severe	Yes	NR	Pneumonia	CT scan	175 ± 20 days	Lung fibrotic-like changes in 35% patients, while in 65% patients complete radiological resolution (38%) or residual ground-glass opacification or interstitial thickening (27%)
Heiss et al. (25)	Case-reports	Erlangen, Germany	•1 male • 60-year-old	Severe	Yes	No	Peripheral, multilobar areas of ground-glass Opacity (GGO)	CT scan MRI	90 days	Residual pulmonary changes with patchy, peripheral GGOs, and consolidations
Hu et al. (26)	Retrospective	•Wuhan, China •1 January 2020–28 February 2020	•46 patients: •19 females •27 males •Mean age: 39.17	•36 mild/moderate •10 severe	Yes	NR	Fever, cough, myalgia, fatigue, vomiting, or diarrhea	CT scan	31 days	Lung lesions completely absorbed only in 28.57 % of patients
Latronico et al. (27)	Prospective	•Brescia, Italy •February–June 2020	•59 patients •Median age: 54–64	Critical	Yes	Yes	Acute respiratory distress syndrome	X-ray, spirometry	90–180 days	Chest X-ray and pulmonary function altered in 70% of patients at 3 months; few patients had persisting respiratory symptoms at 6 months
Liang et al. (28)	Prospective	Wuhan, China	•76 patients: •55 females •21 males •Mean age 41.3 ± 13.8	•69 mild/general •7 severe/critical	Yes	9 patients	NR	Standard questionnaire; pulmonary function tests (total lung capacity -TLC, D_LCO_, carbon monoxide diffusion constant (D_LCO_/VA)	90 days	42% of patients with pulmonary function abnormalities
Liao et al. (29)	Retrospective	•Guangzhou, China •January 22,–April 10, 2020	•158 patients: •22 females •88 males •Mean age: 48.0 ± 17.7	•14 mild •110 moderate •34 severe	Yes	3 patients	fever, fatigue, diarrhea, polypnea, anorexia	Peripheral blood analyses (inflammatory cytokines expression), CT scan	60 days	Persistent elevation of IL-6 associated with persistent pulmonary lesions
Manckoundia et al. (30)	Case-report	Dijon, France	49-year-old man	Mild	No	No	Asthenia, fever, dry cough, dysgeusia, headache	General practitioner consult	90 days	Non-inflammatory tracheal hypersecretion
Mo et al. (31)	Cross-sectional	•Guangzhou, China •February–March 2020	•110 patients: •55 females •55 males •Mean age: 49.10	•24 mild •67 moderate •19 severe	Yes	NR	NR	Spirometry, D_LCO_	20 ± 6 days in mild cases; 29 ± 8 days in moderate cases; 34 ± 7 days in severe cases	D_LCO_ anomalies in 47.2% of patients, total lung capacity in 25.0%, forced expiratory volume in 1 s (FEV1) % in 13.6%, forced vital capacity (FVC) % in 9.1%, FEV1/FVC in 4.5% and small airway function in 7.3% of patients
Moreno-Perez et al. (32)	Prospective	•Alicante, Spain •February–April 2020	•277 patients: •47.3% females •52.7% males •Median age: 62.0	•34.3% mild •65.7% severe	182	NR	NR	Spirometry, chest radiology	70 - 98 days	Spirometry alterations present in 9.3% patients, while in radiographs in 18.9%
Ramakrishnan et al. (33)	Retrospective	•Atlanta, USA •April, 2020	•107 patients: •26 males •81 females •Mean age: 55	NR	NR	NR	Fever, cough, smell or taste alteration	Lung auscultation, ECG	30 days	10% of with dyspnea and fatigue
Shah et al. (34)	Prospective	•Vancouver, Canada •March-May 2020	•60 patients: •32% females •68% males •Median age: 67	NR	Yes	NR	Dyspnoea, cough	Pulmonary function testing (PFT), 6 min walk test (6MWT), high-resolution CT of the chest	90 days	More than half of patients with lung function and chest imaging abnormalities
Sonnweber et al. (35)	Prospective	Innsbruck, Austria	•109 patients: •44 females •65 males •Mean age: 58	•22 mild •34 moderate •35 severe •18 critical	87 patients	18 patients	NR	CT scan, serum biomarkers	60 days	Iron deficiency in 30% of patients, anemia in 9%. Increased inflammation markers levels, such as IL-6 and C-reactive protein in anemic patients. 38% of patients with hyperferritinemia associated with severe lung pathologies
Tabatabaei et al. (36)	Retrospective	Kashan, Iran	•52 patients: •20 females •32 males •Mean age: 50.17 ± 13.1	Severe/critical	Yes	11 patients	Fever, fatigue, dyspnea, GGO, consolidation, and mixed pattern	CT scan, serum biomarkers	90 days	42.3% with residual pulmonary disease. General poor health status in the domains of functional impairment (64%), fatigue (69%), QoL (72%)
Trinkmann et al. (37)	Prespective	•Heidelberg, Germany •March–June 2020	246 patients: Mean age: 48 ± 15	Mild to- severe	20 patients	2 patients	Olfactory loss, cough, pyrexia, dyspnoea, sore throat, rhinitis, thoracic pain, limb pain, cephalgia, fatigue	Spirometry and body-plethysmography	68 ± 16 days	Lower lung function even in younger SARS-CoV-2 convalescents with few comorbidities
Truffaut et al. (38)	Retrospective	•Brussels, Belgium •March–June 2020	•22 patients: •6 females •16 males •Mean age: 54.6 ± 10.9	Severe	Yes	Yes	NR	Pulmonary function test (PFT), 6-min walking distance test (6MWDT), dyspnoea (modified Medical Research Council (mMRC)	90 days	55% of patients with restrictive pattern ± altered D_LCO_. 65% with a 6MWDT below 80% and 52% were free from exertional dyspnoea according to mMRC scale
van den Borst et al. (39)	Prospective	•Nijmegen, The Netherlands •23 April - 15 July 2020	•124 patients •50 females •74 males •Mean age: 59 ± 14	•27 mild •51 moderate •26 severe •20 critical	Yes	Yes	NR	CT scan Clinical Frailty Scale (CFS) Pulmonary function tests (D_LCO_, TLC)	90 days	90% of patients with residual pulmonary parenchymal abnormalities
van Gassel et al. (40)	Retrospective	March–May 2020	48 patients	Severe	Yes	Yes	Severe pneumonia	Pulmonary function testing (PFT), i.e.spirometry, lung volumes, D_LCO_ adjusted for Hb, chest high-resolution CT (HRCT)imaging, and 6-minute-walk test (6-MWT)	90 days	Reduced total lung capacity and diffusion capacity in 23 and 36 participants, respectively, but no airway obstruction on PFT. Ground-glass opacities in 89% of cases. Signs of reticulation, bronchiectasis, bronchiolectasis in 67% of cases
Weerahandi et al. (41)	Prospective	•New York, USA •April 15, 2020,	•152 patients: •57 females •95 males •Mean age: 62	Severe	Yes	101	NR	Patient-Reported Outcomes Measurement Information System (PROMIS®) Dyspnea Characteristics instrument	30–40 days	Shortness of breath in 74% of patients; 35.1% patients require home oxygen after hospital discharge
Yao et al. (42)	Case-report	•China •January 27, 2020	•1 female • 78-year-old	Mild	Yes	No	Multiple patchy shadows in both lungs	Lungs biopsy	14 days	Diffuse alveolar damage, extensive desquamation of proliferative type II alveolar epithelial cells, exudative monocytes and macrophages
Zhao et al. (43)	Retrospective	•3 tertiary hospitals of Henan Province, China •20 January−24 February 2020	•55 patients: •23 females •32 males •Mean age: 47.74	•4 mild •47 moderate •4 severe	Yes	NR	Gastrointestinal symptoms, headache, fatigue, dyspnea, cough, sputum, olfactory, and gustatory dysfunctions	CT scan, pulmonary function test	90 days	Abnormalities of pulmonary function and chest radiography in three quarters of patients. Higher D-dimer level at admission predict impaired D_LCO_ 3 months after discharge
Zhu et al. (44)	Case-report	•Hubei, China •January 2020	30-year-old male	Severe	Yes	NR	Dry cough, fever, emphysema in both upper lungs, with ground glass density at the edge	Chest CT, laboratory examination results, lung function examination, sleep monitoring, sex hormones, sperm morphology and activity	11 months	Abnormal airway function, cough, chest pain, chest tightness, and shortness of breath, unstructured sleep apnea hypopnea syndrome, and nocturnal sleep hypoxemia
***Persistent neurological symptoms and olfactory dysfunctions***										
Boscolo-Rizzo (45)	Prospective	Treviso, Italy	183 patients	Mild	NR	No	Fever, dry cough or coughing up mucus, loss of appetite, felt tired, altered sense of smell or taste	Interviews	60 days	18.6% of patients with altered sense of smell or taste
Caronna et al. (46)	Prospective	•Barcelona, Spain •28 March−22 April 2020	•130 patients: •66 females •64 males •Mean age: 53.9	Mild-to-severe	80%	8.5%	Headache, fever, malaise, myalgia, dizziness, cough, dyspnea, chest pain, expectoration, odynophagia, loss of smell/taste, diarrhea	Neurological assessment	45 days	74.6 % of patients had headache. At follow-up 37.8% of these had persistent headache (50% with no previous headache history)
D'Ascanio et al. (47)	Case-control	•Santa Croce Hospital AORMN, Fano-Pesaro, Italy •1 February–April 24, • 2020	•43 COVID-19 patients •25 healthy controls	Mild	20 patients	No	Anosmia, hyposmia, headache	A 7-question survey instrument, subjective olfactory dysfunction	30 days	Resolution of anosmia or hyposmia in ~85% of patients
Dani et al. (48)	Case-series	London, UK	•6 female patients •Age: 26–50 years	NR	No	No	Gastrointestinal symptoms, upper respiratory tract symptoms, chesty cough, flu-like symptoms	Echocardiogram	21 days	Orthostatic intolerance syndromes (orthostatic hypotension, vasovagal syncope, postural orthostatic tachycardia syndrome)
Fjaeldstad et al. (49)	Retrospective	•Denmark •22 April−4 May 2020	•109 patients: •79 females •30 males •Mean age: 39.4	Mild	No	No	Fever, headache, fatigue, dyspnea, cough sputum, olfactory, gustatory loss	Subjective chemosensory function	> 30 days	28% and 20% of patients not experienced improvement respectively of their olfactory and gustatory function, whereas 44% and 50% fully recovered olfactory and gustatory loss respectively
Galal et al. (50)	Cross-sectional	•Aswan, Egypt •18 July−31 August 2020	•430 patients: •274 females •156 males •Mean age: 37.4 ± 12.6	Mild-to-critical	103 patients	20 patients	Myalgia, fever, restriction of daily activities, memory loss	A 4-point Likert scale	30 days	Myalgia (60.0%), arthralgia (57.2%), restriction of daily activities (57.0%), sleeping troubles (50.9%), nervousness and hopelessness (53.3%), anorexia (42.6%), chest pain (32.6%), gastritis (32.3%), cough (29.3%) and dyspnea (29.1%)
Gallus et al. (51)	Retrospective	•Sassari, Italy •April-May 2020	•48 patients: •37 females •11 males •37 (77%) Mean age: 45	Mild	No	No	Fever, dyspnea, cough, thoracic pain, asthenia, myalgia, diarrhea, conjuntivitis, general malaise, sore throat, headache, cutaneous rash, hypo-anosmia, hypo-ageusia	Tonal pure tone audiometry, a vHIT and SHIMP test	14 days from the second negative swab	8.3% patients reported hearing loss, 4.2% tinnitus, 8.3% dizziness, 2% spinning vertigo, 2% dynamic imbalance, 6.3 static imbalance
Guedj et al. (52)	Case-report	Marseill, France	•54-year-old man •62-year-old man	•1 severe •1 moderate	Yes	Yes	Acute respiratory distress syndrome, anosmia or ageusia	Whole-body^18^F-FDG PET	30 days	Hypometabolism of the olfactory/rectus gyrus on the two patients
Hellmuth et al. (53)	Case-report	San Francisco, CA, USA	•33-year-old woman •56-year-old woman	Mild	No	No	Neck pain, fatigue, fever, cough, myalgias, and non-migrainous headaches, cognitive symptoms	Cerebrospinal fluid and blood analyses, MRI	•149 days •72 days	•Deficits in working memory and digit span backwards with high average attentional skills •Word finding difficulties, inefficient learning, and decreased organization leading to missed deadlines
Lim et al. (54)	Case-report	UK	55-year-old woman	Mild	Yes	No	Fever, myalgia, cough, breathlessness, anosmia, ageusia, headache	CT scan, MRI, Addenbrooke's Cognitive Examination-III	52 days	Persistent psychotic symptoms
Lu et al. (55)	Prospective	•Fuyang No.2 People‘s Hospital, China •January -February 2020	•60 patients: •26 females •34 males •Mean age: 44.10 •39 age and •sex-matched non COVID-19 controls	•47 mild •12 severe •1 critical	Yes	NR	Fever, cough, gastrointestinal symptoms, neurological symptoms	Diffusion tensor imaging (DTI), 3D high-resolution T1WI sequences	90 days	68.33% of patients with disruption to micro-structural and functional brain integrity during infection and 55% of them maintain the same symptoms after 90 days
Mendez et al. (56)	Prospective	•Valencia, Spain •March -April	•179 patients •74 females •105 males •Mean rage: 22–81	Mild-to severe	Yes	34 patients	NR	Standardized instruments evaluating neurocognitive function, psychiatric morbidity, and QoL	60 days	58.7% presented at least moderate neurocognitive decline, 39.1% psychiatric morbidity, and ~40% had poor QoL
Moein et al. (57)	Prospective	•Tehran, Iran •21 March−3 May, 2020	•82 patients: •28 females •45 males •Mean age: 45.53	•58 mild •30 moderate •12 severe	Yes	No	Fever, cough, breathlessness, headache, myalgia, shivering, sweating, gastrointestinal symptoms, malaise, tinnitus, bloody sputum	40-item University of Pennsylvania Smell Identification Test (UPSIT)	40–60 days	96% of patients with smell loss during infection. At follow-up, the test scores of 63% of the retested patients were normal. However, the mean UPSIT score at that time continued to remain below that of age- and sex matched healthy controls
Negrini et al. (58)	Case-series	•Milan, Italy •3 March−8 April, 2020	•9 patients: •3 females •6 males •Mean age: 60	•4 mild/moderate •5 severe	Yes	5 patients	NR	Mini-Mental State Examination (MMSE) test	30 days	General cognitive decay in 33.3% of patients, with a specific decline in attention, memory, language, and praxis abilities. The cognitive decay appears to be associated with the length of stay (in days) in ICU
Novak et al. (59)	Case-report	Boston, USA	64-year-old woman	NR	No	No	Cough, dyspnea	CT scan	20 days	Probable orthostatic hypoperfusion syndrome and painful small fiber neuropathy in post- COVID disease.
Panda et al. (60)	Prospective	•New Delhi, India •23 April−29 June 2020	•225 patients: •63 females •159 males •3 transgenders •Mean age: 34.96	•145 mild •80 asymptomatic	No	No	Otolaryngologic symptoms, fever, cough, dyspnea, gastrointestinal symptoms	Ear, Nose and Throat (ENT) symptoms evaluation	28 days	96% of the patients regaining ENT function at follow-up
Pilotto et al. (61)	Retrospective	•Brescia, Italy •February–April 2020	•165 patients: •50 females •115 males •Mean age: 64.8 ± 12.6	Moderate-to severe	Yes	NR	NR	Montreal Cognitive Assessment (MoCA) score	180 days	Fatigue (34%), memory/attention (31%), sleep disorders (30%). 37.4% of patients with neurological abnormalities, i.e. cognitive deficits (17.5%), hyposmia (15.7%), postural tremor (13.8%)
Pritza et al. (62)	Retrospective	•Thessaloniki, Greece •March–April 2020	•90 patients: •37 females •53 males •Mean age: 55.8 •± 17.3	•45 mild •35 moderate •10 severe	Yes	10 patients	Olfactory and gustatory dysfunction	Questionnaires	61 days	8.57 % patients with persistent hyposmia
Raahimi et al. (63)	Case-report	Portsmouth, UK	46-year-old man	Severe	Yes	Yes	Sensory loss in his feet, progressing to gait unsteadiness and distal lower limb weakness	Cerebrospinal fluid analysis, ECG, CT scan, MRI, spirometry	90–150 days	At 90 days intermittent neuropathic pain and paraesthesia in distal limbs were present. At 150 days improvement in nerve function, with normalizing distal motor latencies
Sampaio Rocha-Filho et al. (64)	Case-report	Recife, Brazil	40-year-old woman	Mild	No	No	Diarrhea, cough, fatigue, myalgia, anosmia, facial pain, headache	MRI, intracranial magnetic resonance angiography	85 days	Persistent anosmia and headaches
Tobechukwu et al. (65)	Case-report	Red Bank, USA	46-year-old woman	Mild	Yes	No	Fever, chest pain, vomiting, cough, confusion	X-ray, CT scan. MRI	90 days	Delirium and allucinations
Ugurlu et al. (66)	Retrospective	•Çorum, Turkey •March–June 2020	•42 patients: •23 female 19 male Mean age: 41.2 ± 14.6	Mild	No	No	Fever, cough, dyspnea, diarrhea, sore throat, nasal drip, nasal obstruction, headache	Brief smell identification test	90 days	Full recovery in 85.7% of patients. Olfactory dysfunction persisted in 14,3% of patients
Vaira et al. (67)	Prospective	•University •Hospital of Sassari, San Paolo Hospital in Milan, and Bellaria-Maggiore Hospital in •Bologna, Italy	•138 patients: •70 females •68 males •Mean age: 51.2	Mild-to-severe	Yes	NR	Chemosensitive dysfunction	Self-administered olfactory and gustatory psychophysical tests in outpatients, Connecticut Chemosensory Clinical Research Center orthonasal olfaction test in hospitalized patients	60 days	5.8 % with moderate to severe olfactory dysfunction, 4.3 % with significant taste disorder. Four patients with combined chemosensitive dysfunctions, 4 patients with isolated smell impairments and two patients with isolated taste disorders
Yan et al. (68)	Cross-sectional	•California, USA •9 March–April 29, 2020	46 patients	NR	NR	NR	NR	10-point scale score for sense of smell	16 days	Olfactory dysfunction reported by 23 patients (17 reported no loss, 5 were unreachable, 1 died). At follow up 78% of patients with chemosensory dysfunction
***Widespread persistent symptoms***										
Abdallah et al. (69)	Case-report	•Philadelphia, USA •March 2020	30-year-old man	Mild	No	No	Chest pain, fever, anosmia	X-ray, CT scan	8 months	Chest pain, dyspnoea, and fatigue, intercostal neuralgia
Arnold et al. (70)	Prospective	•Southmead Way, Bristol •30 March and 3 June 2020	110 patients	27 mild65 moderate18: severe	Yes	No	NR	Chest radiograph, spirometry, exercise test, bloods, and health-related quality of life (HRQoL) questionnaires	83 days	Most (74%) patients with persistent symptoms (notably breathlessness and excessive fatigue) with reduced HRQoL
Buonsenso et al. (71)	Cross-sectional	•Rome, Italy •March–November 2020	•129 children: •62 females •67 males •Mean age: 11 ± 4.4	Mild-to severe	6 patients	3 patients	NR	Questionnaire	162.5 ± 113.7 days	35.7% had 1 or 2 symptoms and 22.5% had 3 or more. 52.7% had at least one symptom 120 days or more after diagnosis. Fatigue, muscle and joint pain, headache, insomnia, respiratory problems and palpitations are the main reported symptoms
Buselli et al. (72)	Case-report	Azienda Ospedaliero-Universitaria Pisana, Pisa, Italy	50-year-old woman	Mild	No	No	Dry cough, asthenia, myalgia, diarrhea, fever, dyspnea, headache, fatigue, dysphonia	Pneumology examination, CT scan, neurological examination with brain scan, cardiology examination with echocardiograph, pulmonary ultrasound and ENT specialist examination	≥ ys	Persistent fatigue and dysphonia
Carfi et al. (73)	Retrospective	•Fondazione Policlinico Universitario •Agostino Gemelli IRCCS, Rome, Italy •21 April – 29 May, 2020	•143 patients: •53 females •90 males •Mean age: 56.5	•21 mild •104 moderate •18 severe	Yes	18 patients	Fatigue, dyspnea, joint pain, chest pain, cough, anosmia, sicca syndrome, rhinitis, red eyes, dysgeusia, headache, sputum production, lack of appetite, sore throat, vertigo, myalgia, diarrhea	EuroQol visual analog scale	60 days	At follow-up, only 12.6% of patients with no COVID-19 related symptom, while 32% had 1 or 2 symptoms and 55%had 3 or more. Main persistent symptoms were fatigue (53.1%), dyspnea (43.4%), joint pain, (27.3%) and chest pain (21.7%).
Carvalho-Schneider et al. (74)	Prospective	•Tours University Hospital, France •17 March−3 June, 2020	•150 patients: •84 females •66 males •Mean age: 49	Non-critical	Yes	No	Dyspnea, fever, weight loss, chest pain, headache, asthenia, myalgia, gastrointestinal symptoms, anosmia, ageusia	Clinical algorithm	30 and 60 days	At 30 days 68% of patients with at least one symptom and 66% at 60 days. Anosmia/ageusia: 28% at 30 days, 23% at 60 days. Dyspnea: 36.7% at 30 days, 30% at 60 days. Asthenia: 50% at 30 days, 40%) at 60 days. Persistent symptoms at 60 days significantly associated with age 40–60, hospital admission and abnormal auscultation at symptom onset
Chen et al. (75)	Cross-sectional	•12 Hospitals in Wenzhou, Zhejiang, China •17 January−20 March, 2020	•361 patients: •175 females •186 males •Mean age: 47.22	•327 mild •34 severe	Yes	NR	NR	Chinese version of Short-Form 36-item questionnaire (SF-36)	30 days	Health-related quality of life (HRQoL) was poor among COVID-19 patients at follow-up
Cirulli et al. (76)	Prospective	•Nevada, USA •April–September 2020	233 patients	Mild	8 patients	No	Fever, headache, asthenia, fatigue, diarrhea, ageusia, dry cough, chest pain, bone and joint pain, red eyes, dizziness, anorexia	Self-reported short and long-term symptoms	30 and 90 days	43.4% of patients with symptoms longer than 30 days, 24.1% with at least one symptom after 90 days. Long-term symptoms were anosmia, ageusia, difficulty concentrating, fatigue, dyspnea, memory loss, confusion, headache, heart palpitations, chest pain, pain with deep breaths, dizziness, and tachycardia
D'Cruz et al. (77)	Prospective	June–July 2020	•119 patients: •45 females •74 males •Mean age: 58.7 ± 14.4	Severe	Yes	Yes	Pneumonia	X-ray, CT scan, clinical outcomes, symptom questionnaires, mental health screening, physiologic (4MGS and STS) al testing	51–67 days	Persistent fatigue (68%), sleep disturbance (57%) and breathlessness (32%), post-traumatic stress disorder (25%), anxiety (22%) and depression (18%). 4MGS was slow in 38% and 35% desaturated by ≥4% during the STS test
Erçalik et al. (78)	Retrospective	•Istanbul, Turkey •March–May 2020	•206 patients: •105 females •101 males •Mean age: 56.24 ± 16.99	•153 mild •48 moderate •5 severe	Yes	Yes	Fever, cough, dyspnea, runny nose	Pain assessment using a numeric rating scale	45.99 ± 14.64 days	40.7% of the patients had chronic pain for at least 3 months before COVID, and this rate increased to 82.5% during COVID and to 55.1% after COVID
Galván-Tejada et al. (79)	Case-control	•Zacatecas Mexico •July–September!!!!2020	•219 patients: •141 recovered •78 controls •51% females •49% males •Mean age female: 39.14 Mean age male: 39.01	NR	Yes	NR	NR	Questionnaire	60 days	Chills, dyspnea, anosmia or dysgeusia, nausea or vomiting cough, red eyes as persistent symptoms in COVID-19 patients
Hosseini et al. (80)	Case-report	Qom, Iran	48-year-old man	Mild	No	No	Fever, chills, weakness, lethargy, myalgia	Laboratory Tests, CT scan, ECG	30 days	Persisten advanced atrioventricular block
Huang et al. (81)	Prospective	Wuhan, China between January−7 May 2020	•1733 patients •48% females •52% males •Median age: 57.0	Mild to-severe	Yes	76 patients	NR	Questionnaires, physical examination, blood tests, CT scan, 6-min walking test	186 days	Fatigue or muscle weakness (63%), sleep difficulties (26%) were the most common symptoms. Anxiety or depression was reported among 23% of patients
Isoldi et al. (82)	Prospective	•Latina, Italy •April–June 2020	•15 children: •7 females •8 males •Median age: 12.2	Mild	No	No	Fever, hyperemia of the pharynx (53.3%), abdominal swelling, tender to the touch (33.3%), active conjunctival injection (6.7%)	Laboratory (blood, urine, feces) tests, ECG	180 days	Two patients with hyperfiltration exhibited high blood pressure levels at diagnosis, and persistence of a prehypertension at 6-month follow-up
Iqbal et al. (83)	Cross-sectional	•Karachi, PAK •September–December!!!! 2020	•158 patients: •87 females •71 males •Mean age: 32.10 ± 12.42	•112 mild •33 moderate •13 severe	Yes	13 patients	NR	Questionnaire	20–90 days	Fatigue (82.9%), poor sleep quality (56.3%), anxiety (53.2%), dyspnea (50%), joint pain (47.5%) were the most prevalent post-discharge manifestation
Jacobs et al. (84)	Prospective	•New Jersey, USA •22 March–April 16	•183 patients •38.5% females •61.5% males •Mean age: 57 •61.5% male	•160 mild •23 severe	Yes	23	Fatigue, shortness of breath, cough, lack of taste, muscular pain, diarrhea, lack of smell, production of phlegm, headache	PROMIS® instruments to identify symptoms and quality of life parameters	35 days	Fatigue (55.0%), dyspnea (45.3%), muscular pain (51%), lower odds rating general health (41.5%), quality of life (39.8%), physical health (38.7%), mental health (43.7%) and social active role (38.7%)
Khalaf et al. (85)	Cross-sectional	•Assiut, Egypt •August–October 2020,	•538 patients •Mean age: 41.17 ± 14.84 •45.9 females •54.1% males	•Mild-to-severe •(61.3% mild, 31% moderate, 7.6% severe)	51.3% of patients	6.5% of patients	NR	Online questionnaire	83 days	Fatigue (59.1%), sense of fever (46.5%), anorexia (24.3%), diarrhea (24.3%), loss of taste and smell (22.3%), headache (21.4%), cough (20.8), dyspnea (21%)
Ludvigsson et al. (86)	Case-report	•Stockholm, Sweden •October 2020	•5 children •Mean age: 12 •4 girls •1 boy	Mild	No	No	Fever, dyspnea, abdominal pain, upper respiratory symptoms, dizziness, extreme fatigue, cough, lost taste and smell, headache, abdominal pain, diarrhea, nausea, norexia	NR	6–8 months	Fatigue, dyspnoea, heart palpitations or chest pain, headaches, difficulties concentrating, muscle weakness, dizziness, sore throats
Mandal et al. (87)	Cross-sectional	London, UK	•384 patients •Mean age: 59.9 •38% females •62% males	Mild-to-critical	Yes	54 patients	NR	CT scan, blood tests, 11-point (0–10) scale score	54 days	Persistent breathlessness (53%), cough (34%) fatigue (69%), depression (14.6%), elevated d-dimer (30.1%) and C reactive protein (9.5%), abnormal chest radiographs (38%)
Mahmud et al. (88)	Prospective	•Dhaka, •Bangladesh •June–August 2020	•355 patients •148 females •207 males •Mean age: 39.8	•221 mild •93 moderate 41 severe	Yes	Yes	Fever, cough, respiratory distress, anosmia, anorexia headache, lethargy	Telephonic interview	At least 30 days	46% of patients developed long-term symptoms. Post-viral fatigue (70%) was the most prevalent symptom. Post-COVID features are significantly higher among female
Martin et al. (89)	Retrospective	•USA •March–September!!!! 2020	9,989 patients	Mild- to severe	Yes	NR	NR	Electronic health records	90–180 days	Persistent neuropsychiatric, pulmonary, metabolic, and coagulopathic phenotypes
Pellaud et al. (90)	Retrospective	•Fribourg, Switzerland •March–April 2020	•196 patients: •77 females •119 males •Mean age: 70	•Mild •Moderate •Severe/Critical	Yes	49 patients	NR	Data collected by electronic health records or by telephone	30 days	Among 117 patients discharged from hospital within 30 days after the beginning of symptoms, 63% reported persistent symptoms. The main persistent symptoms are asthenia (67%), respiratory symptoms (56%), anosmia/dysgeusia (10%)
Petersen et al. (91)	Retrospective	•Tórshavn, Faroe Islands •22 April−16 August 2020	•180 patients •Mean age: 39.9 ± 19.4 •98 females •82 males	Mild-to-moderate	8 patients	No	Fatigue, fever, headache, chills, and loss of smell and taste	Questionnaire	125 days	53.1% reported persistence of at least one symptom, 33.3% reported one or two symptoms and 19.4% three or more symptoms. Most prevalent persistent symptoms: fatigue, loss of smell and taste, arthralgias
Raman et al. (92)	Prospective	Oxford, UKMarch–May 2020	•58 patients: •24 females •34 males •Mean age: 55 ± 13	Moderate to- severe	Yes	21 patients	Fever, malaise, shortness of breath, cough, dysgeusia, anosmia, diarrhea, chest pain, headache, vomiting	MRI of the brain, lungs, heart, liver, kidneys, 6-minute walk (6MWT) test, spirometry, cardiopulmonary exercise test (CPET), questionnaires, blood tests	60–90 days	•64% of patients experienced breathlessness and 55% fatigue. MRI, abnormalities in lungs (60%), heart (26%), liver (10%), and kidneys (29%). •Impaired cognitive performance and reduced six-minute walk distance
Rosales-Castillo et al. (93)	Retrospective	March–May 2020	•118 patients: •44.1 females •55.9% male •Mean age: 60.16	Mild to-severe	Yes	7.6% of patients	Fever, cough, dyspnoea, diarrhea, ageusia, myalgia, anosmia, chest pain, headache, expectoration	Physician consultation	50 days	62.5% of patients reported persistence of symptoms: dyspnoea (31.4%), asthenia (30.5%), myalgia (13%), cough (5%), anosmia (1.7%), and ageusia (1%)
Saeed et al. (94)	Case-report	Lahore	•48-years-old- woman •42-years-old- woman •32-years-old- woman •37-years-old- woman	Mild	No	No	Dry cough, fever, abdominal discomfort and diarrhea	Dermatological consulting	60–90 days	Hair shedding: telogen effluvium
Saiful Islam et al. (95)	Cross-sectional	•Bangladesh •September–October 2020	•1,002 patients: •422 females •580 male •mean age = 34.7 ± 13.9	Mild to- severe	208 patients	NR	Fever and fatigue	Online questionnaire	30 days	20% of patients reported persistent symptoms. The most reported persistent symptoms were diarrhea (12.7%) and fatigue (11.5%). 48% of participants had moderate to severe depression
Smane et al. (96)	Retrospective	•Riga, Latvia •July 2020	•30 children: •13 females •17 male •Mean age: 9.2	•5 asymptomatic •24 mild •1 moderate	No	No	Fever, rhinorrhoea, cough	Physician assessment	101 days	70% patients completely free of any COVID-19-related symptoms, while 30% had at least one symptom (fever, joint pain, headache, anosmia, ageusia, microhaematuria)
Sofian et al. (97)	Case-series	•Arak, Iran •February–April 2020	•10 patients •9 females •1 male	Mild-to moderate	No	No	Fever, dry cough, nasal congestion, weakness, high diaphoresis, loss of smell, fatigue	CT scan	60 days	Dry cough, headache, severe sweating, shivering, loss of smell, mild on/off fever, and diarrhea, weight loss
Stavem et al. (98)	Cross-sectional	•Lørenskog, Norway •Until 1 June 2020	•451 patients: •253 females •198 males •Mean age: 49.7	Mild	No	No	Fever, loss of smell, headache, dry cough, myalgia, chills, dyspnea, sore throat, gastrointestinal manifestations	Mixed-mode survey	117 days	53 % of woman and 67 % of men with no persistent symptoms. Fatigue and dyspnoea are common about 60 days
Sykes et al. (99)	Retrospective	Hull, UK	•134 patients: •46 females •88 males •Median age: 58	Mild to-severe	Yes	20% patients	Breathlessness, myalgia anxiety, fatigue, low mood, sleep disturbance	X-ray, standardized clinical assessment, questionnaires for dyspnea, and quality of life	113 days	86% of patients reported at least one residual symptom: breathlessness (60%), anxiety (47.8%), extreme fatigue (39.6%), lowmood (37.3%), and sleep disturbance (35.1%). Females reported most residual symptoms including anxiety, fatigue, and myalgia
Taboada et al. (100)	Prospective	•Santiago, Spain •March–April 2020	•91 patients: •32 females •59 males •Mean age: 65.5	Critical	Yes	All patients	Myalgia, asthenia, insomni, arthralgi, cough, anosmia, chest pain	Questionnaire	180 days	Decrease in quality of life in 67% of patients (56% mobility, 37% usual activities, 13% self-care, 48% pain/discomfort, 46% anxiety/depression). Dyspnoea on exertion (57%), asthaenia (37%), myalgia (37%), and arthralgia (29%). Only 16% of patients were completely free of persistent symptoms
Townsend et al. (101)	Retrospective	Dublin, Ireland	•128 patients •54% females •46% males •Mean age: 49.5 ± 15	Mild-to critical	71 patients	18 patients	Fatigue	Chalder Fatigue Score (CFQ-11), markers of peripheral immune activation and circulating pro-inflammatory cytokines	72 days	52.3% of patients reported persistent fatigue. No association between fatigue and COVID-19 severity, laboratory markers of inflammation, pro-inflammatory molecules
Townsend et al. (102)	Cross-sectional	•Dublin, Ireland •March–May 2020	•153 patients: •57 females •96 males •Median age: 48	Mild-Critical	Yes	19 patients	NR	X-ray	75 days	Persistent abnormal x-rays of either persistent infiltrate or atelectasis in 19% of patients. 62% patients had not returned to full health, while 47% met the case definition for fatigue
Varghese et al. (103)	Retrospective	•Münster, Germany •June–September 2020	•116 patients •17 females •99 males •Mean age: 41	NR	10 patients	No	Cough, anosmia, fatigue, fever, myalgia, headache	Laboratory measurements, attending physicians document symptoms	22–102 days	•At 3 months of follow-up persisting symptoms were fatigue (54%), dyspnea (29%), and anosmia (25%), lymphopenia (12%) •Lymphopenia in the later follow-up range of 80–102 days
Ya-Wen An et al. (104)	Cross-sectional	•Guangdong, China •February–May 2020	•46 patients: •20 females •26 males •Mean age: 46.8 ± 15.3	•36 non-severe •10 severe	Yes	Yes	Fever, weak blocked or watery nose haryngeal symptoms muscle or joint pain chest distress dizziness or headache gastrointestinal symptom	Blood routine, blood biochemistry, urine routine, stool routine, and chest CT scans	60 days	Extremely low outlier ratio of total protein, albumin, and globulin

### Inclusion and Exclusion Criteria

Papers of any design evaluating individual persistent symptoms in mild, moderate, severe, and critical COVID-19 patients that have a follow-up of at least 14 days were included in this review.

Exclusion criteria included: unpublished reports, unspecified date/location of the study or suspicion of duplicate reporting, coronavirus strains other than COVID-19, unreported long-term COVID-19 symptoms, and studies that only hypothesize post-COVID-19 sequelae.

### Study Selection and Data Extraction

Possible relevant articles were screened using the title and abstract by one reviewer (FS) and articles that did not meet the inclusion criteria were excluded. After screening the title and abstract, articles were submitted to a public reference manager (Mendeley v.1.17.9) to eliminate duplicates. Subsequently, the remaining full-text articles were examined by two reviewers (FS and FV). Any disagreement was resolved through discussion until a consensus was reached, or with the involvement of a third reviewer (MF).

The following items were extracted from each cohort study, cross-sectional, case-report, cases-series, case-control studies, if available: author, study type, study country, and period, patient characteristics (numbers, gender, age), COVID-19 severity (mild, moderate, severe, and critical), hospitalization, ICU admission, baseline COVID-19 symptoms, method of evaluating long-term COVID-19 symptoms, follow-up, and long-term COVID-19 symptoms.

### Risk of Bias Assessment

Two reviewers (FS and FV) independently assessed the methodological quality of cohort, cross-sectional, case-reports, case-control, case-series studies, and reviews. Disagreements regarding the methodological quality of the studies were discussed between the two reviewers. If consensus was not reached, a third reviewer (MF) arbitrated. Cohort and Cross-Sectional Studies were assessed by Quality Assessment Tool for Observational Cohort and Cross-Sectional Studies from the National Heart, Lung, and Blood Institute (NIH) (16). Case-control studies were assessed by the quality assessment criteria of The Quality Assessment Tool for Case-Control Studies from NIH (16). The methodological quality of case-series and case-reports were assessed by the quality assessment tool proposed by Murad et al. (17). Finally, reviews were assessed by the Quality Assessment Tool for Systematic Reviews and Meta-Analyses from NIH (16). No bias evaluation was performed for letters, commentary, editorial, news articles, survey, practice, communications, and medical hypothesis.

## Results

### Study Selection and Characteristics

**Figure 1:**
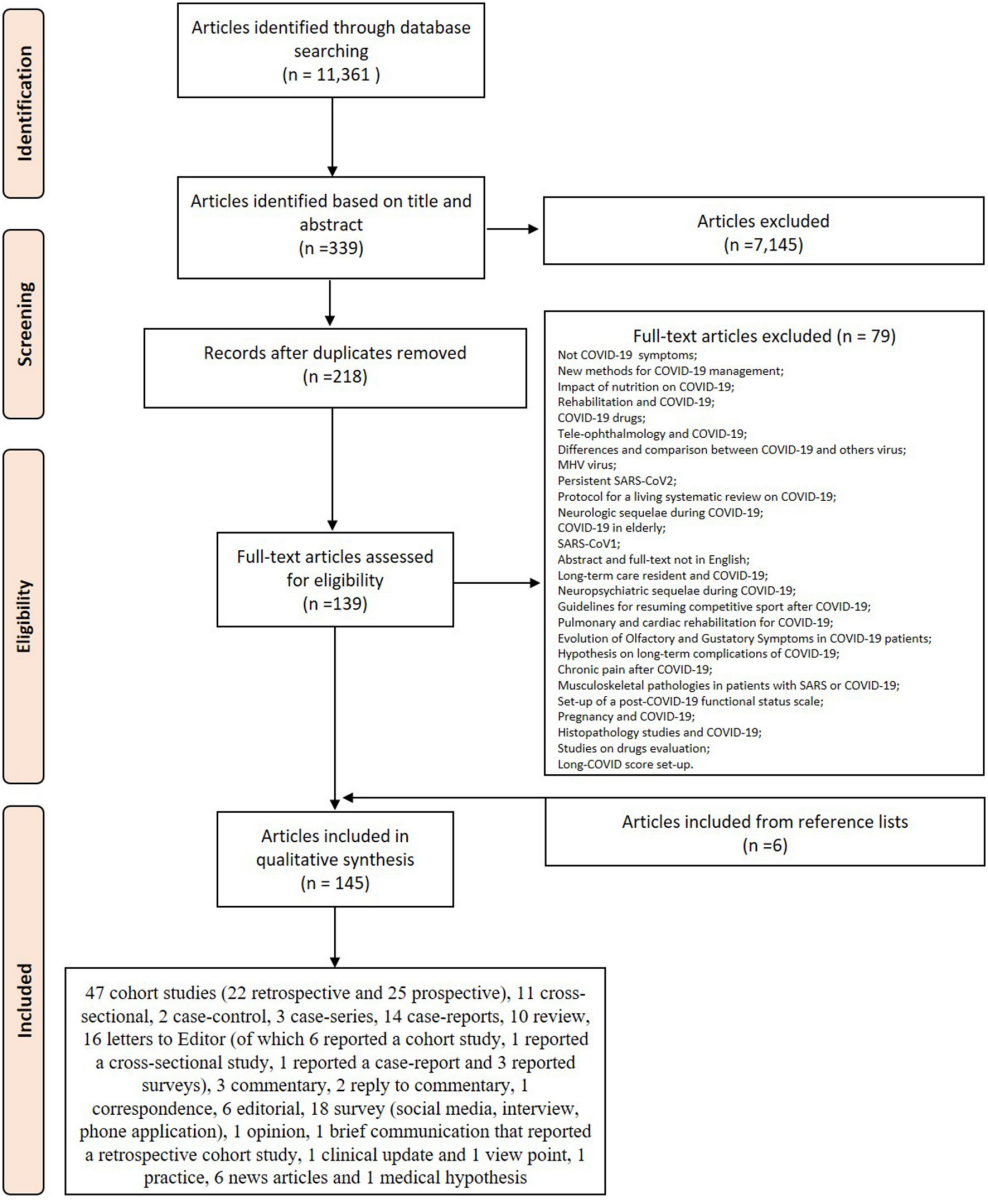
PRISMA flowchart for the study selection.

The initial literature search retrieved 11,361 studies. Of those, 3,132 studies were identified using PubMed, 2,776 using Web of Science, 2,073 using EMBASE and 3,380 using Google Scholar. After screening the title and abstract 315 articles were run through Mendeley to eliminate duplicate articles. The resulting 218 full-text articles were then reviewed to establish whether the publication met the inclusion criteria and 139 were considered eligible. From the reference lists of the selected articles 6 additional publications were found. Of the 145 articles eligible for this review 47 were cohort studies (22 retrospective and 25 prospective), 11 cross-sectional, 2 case-control, 3 case-series, 14 case-reports, 10 review, 16 letters to Editor (of which 6 reported a cohort study, 1 reported a cross-sectional study, 1 reported a case-report, and 3 reported surveys), 3 commentary, 2 reply to commentary, 1 correspondence, 6 editorial, 18 survey (social media, interview, phone application), 1 opinion, 1 brief communication that reported a retrospective cohort study, 1 clinical update and 1 view point, 1 practice, 6 news articles, and 1 medical hypothesis. Search strategy and study inclusion and exclusion criteria are detailed in Figure 1.

### Risk of Bias Assessment

Of the 145 articles eligible for the review, we found 54 cohort studies (28 prospective, 26 retrospective), six of which were published as letters and one as brief communication, and 12 cross-sectional studies, one of which was published as letters. Using the NIH Quality Assessment Tool for Observational Cohort and Cross-Sectional Studies (16), we rated three prospective studies, one ambidirectional cohort study and two cross-sectional studies at a “good” quality rating and 60 studies at a “fair” quality rating (Supplementary Material 1). For the 60 cohort and cross-sectional studies at a “fair” quality rating, the principal missing quality assessment criteria were sample size justification, blinded assessors to the exposure of participants, and missing data on key potential confounding variables measured and adjusted statistically for their impact on the relationship between exposure(s) and outcome(s). Concerning the two case-control studies found, one was at a “good” quality rating and it did not specify only if outcome assessors did not know whether participants were exposed/unexposed, while the other was at a “fair” quality rating (Supplementary Material 1). For the case-control at “fair” quality rating, data on sample size justification, random selection of cases and controls, measures of exposure/risk across all study participants and on blinded assessors of exposure/risk were not reported. The methodological quality of the three case-series and of the 15 case-reports, one of which was published as letters, assessed by the tool proposed by Murad et al. (17), showed that 17/18 studies were at a “good” quality rating (Supplementary Material 1). For the two case-reports rated at a “fair” quality rating, the missing quality assessments criteria were the not adequately ascertained outcome, the lack of alternative causes that may explain the observation, and the absence of sufficient and specific details to describe the case. The quality assessment of reviews showed that 1/10 reviews was at a “good” quality rating while all the others were at a “poor” quality rating (Supplementary Material 1). The “poor” quality rating was because none of them include a comprehensive search of potentially relevant articles and did not use explicit criteria in the selection of articles. The research designs and study characteristics were not appraised, data were not synthesized, and results were not interpreted using a predefined systematic approach.

### Long-Term Symptoms of COVID-19

Of the 145 eligible papers, 30 were on persistent lung symptoms (20.70%), 35 were on persistent neurological and olfactory dysfunctions (24.13%), and 80 were on widespread persistent symptoms (55.17%) (Table 1). Table 1 was split-up based on long-term lung symptoms, long-term neurological and olfactory symptoms, and widespread long-term symptoms.

#### Persistent Lung Symptoms and Dysfunctions

While SARS-CoV-2 was detected in many organ systems, the lungs seem to be the main organs affected by the virus (105–107). Abnormal lung functions and structural changes were reported up to 6 months after hospitalization in mild-to-critical COVID-19 patients (25, 27, 28, 32, 34, 36, 39, 41), also with diffuse alveolar damage, desquamation of alveolar epithelial type II cells, fibrine exudation, hyaline membranes, scattered interstitial inflammation, monocytes, and macrophages (23, 24, 34). Several authors reported that these persistent lung symptoms and dysfunctions correlated with prior COVID-19 severity (19, 20, 24, 25, 27, 36, 38, 41). In this context, Han et al. in a prospective study evaluating 114 severe COVID-19 patients showed lung fibrotic-like changes in 35% patients up to 6 months after infection (24). Differently, Latronico et al. showed that since residual abnormal chest-X ray findings were detected in about 70% of critically ill COVID-19 patients at 3 months, very few of them (~12%) had persisting respiratory symptoms at 6 months (27). An anecdotal

study by Zhu et al. also reported long-term abnormal airway function for up to 11 months in a severe COVID-19 patient (44). However, because is a single case, this research does not provide conclusive evidence. A small cohort of critically ill COVID-19 patients also showed alteration in the diffusing capacity of the lung for carbon monoxide (D_LCO_) for up to 3 months (20, 27, 38, 40). Persistent D_LCO_ impairment was also detected in non-critical COVID-19 patients that also presented shortness of breath and dyspnea up to 4 months after infection (18, 22, 33, 43). Unlike the above cited studies which analyzed exclusively critically ill patients and two case-reports that analyzed solely mild COVID-19 patients (30, 42) all other studies analyzed heterogeneous cohorts of patients, i.e., from mild to severe. Lower lung functions were detected in 246 mild-to-severe SARS-CoV-2 convalescents patients with few comorbidities up to 2 months after infection (37). Widespread lung damages in mild-to-severe COVID-19 patients were further confirmed by numerous papers and by an Editorial where it was underlined that “*months after infection with SARS-CoV-2, some people are still battling lung damage*” (108–110), with more than one-third of them that having pulmonary tissue death and visible scars up to 6 months after symptoms onset (18, 21, 26, 35, 109, 110). In a news feature article it was reported that these lung damages lessened with time, 88% of patients had visible damage up to 6 weeks after infection, but 2 months after symptom onset this number had fallen to 56% (109). By examining retrospectively a cohort of 158 mild-to-severe COVID-19 patients, it was shown that these persistent pulmonary damages were also associated with a persistent elevation of IL-6 up to 2 months after infection (29). At the same follow-up, Chun et al., evaluating 61 prevalently non-critical COVID-19 patients, highlighted also higher levels of Lipocalin 2, suggesting that COVID-19 patients may have an ongoing neutrophil activation that could be amenable to targeted therapy (19). Sonnweber et al., evaluating a cohort of 109 patients with mild-to-critical COVID-19, showed that severe lung pathologies were also significantly associated with persisting hyperferritinemia that was present in ~38% of patients (35). Other authors evaluated the lung abnormalities by CT scans at different stages of SARS-CoV-2 infection (21, 26). Ding et al., analyzing retrospectively a cohort of 112 COVID-19 patients at different stages of the disease, showed that the frequency of crazy-paving pattern, consolidation, and linear opacities peaked at 10–14 days (62.7%), 15–21 days (75.0%), and at 22–28 days (83.1%) and decreased thereafter (21). However, at more than 28 days of follow-up 98.1% of CT scans still showed abnormalities. Similarly, Hu et al., evaluating 46 patients with mild-to-severe COVID-19 who had an isolated pulmonary lesion on the first positive CT, highlighted the presence of reticular patterns from the 14 days after symptoms onset in 45% of patients. At 22–31 days, the lesions were completely absorbed only in 28.57% (26). Mo et al. also noted pulmonary anomalies in a cohort of 110 discharged COVID-19 cases, 24 mild cases, 67 cases of pneumonia and 19 cases of severe pneumonia (31). The duration from onset of disease to pulmonary function test was 20 ± 6 days in mild cases, 29 ± 8 days in pneumonia cases and 34 ± 7 days in cases with severe pneumonia (110). Anomalies were noted in D_LCO_ (47.2%), total lung capacity (25.0%), forced expiratory volume in 1 s (FEV1) (13.6%), forced vital capacity (FVC) (9.1%), FEV1/FVC (4.5%), and small airway function (7.3%) (31).

#### Persistent Neurological Symptoms and Olfactory Dysfunctions

Despite SARS-CoV-2 primarily affecting lungs, numerous data supported the neuro-invading potential of SARS-CoV-2 and, according to the first-hand evidence by Mao et al., ~36.4% of COVID-19 patients presented neurological symptoms (5, 111). Additionally, conditions such as hypoxia, encephalitis, and stroke, all present in severe COVID-19 patients, can produce both long-term neurological symptoms and permanent neurocognitive impairment (52, 58, 112, 113). In fact, a case-series by Negrini et al. associated the long-term neurological symptoms and general cognitive decay to the length of stay in the ICU (58). Despite the long-term neurological symptoms and the general cognitive decay being associated to severe/critical COVID-19 patients, in this review we did not find any studies based solely on critically/severely ill patients. On the other hand, we found a retrospective study and several case reports on mild COVID-19 patients (51, 53, 54, 65). Gallus et al., evaluating retrospectively 48 mild COVID-19 patients, underlined that 8.3% patients reported hearing loss, 4.2% tinnitus, 8.3% dizziness, 2% spinning vertigo, 2% dynamic imbalance, and 6.3% static imbalance at about 1 month of follow-up (51). Several anecdotal reports in mild COVID-19 patients also detected persistent deficits in memory and psychotic symptoms during up to 5 months of follow-up (53, 54, 65). In addition to these studies, all the others found in this review analyzed heterogenic populations of patients with COVID-19, from mild to severe. In this context, a recent editorial and a systematic review provided a detailed overview into the spectrum of mental disorders that can occur during the intermediate and long-term phases of COVID-19 in mild-to-critical patients (114, 115). The most frequent neurological long-term symptoms in these patients were myalgia, arthralgia, sleeping troubles, and headache (46, 50, 61, 116). Additionally, a general cognitive decay, i.e., deficit in attention and calculation, short-term memory, constructional apraxia, and written language, was also observed during up to 6 months of follow-up (61). At 2 months of follow-up 58.7% of 179 mild-to-severe COVID-19 patients presented a moderate neurocognitive decline while 39.1% of patients also showed psychiatric morbidity (56). At a longer follow-up of 6 months, Pilotto et al., analyzing retrospectively 165 moderate-to-severe COVID-19 patients, showed that these long-term symptoms persisted in about 37% of patients (61). Also, symptoms consistent with orthostatic hypoperfusion syndrome and painful small fiber neuropathy were reported at short (3 weeks) and long (up to 3 months) follow-ups in two case-reports and in a small case-series (48, 59, 63). In a “*Long-Haul COVID*” communication, Nath et al., summarizing symptoms reported after mild-to-severe COVID-19, also highlighted persistent symptoms that overlapped with those patients with myalgic encephalomyelitis/chronic fatigue syndrome (117). In addition to the long-term neurological symptoms Lu et al. prospectively examined the presence of brain micro-structural changes in 60 mild-to-critical COVID-19 patients reporting presence of alterations in 50% of recovered patients after 3 months (55). Anecdotal evidence also showed the presence of long-term impairment of the brain structures in two COVID-19 patients highlighting hypometabolism of the olfactory/rectus gyrus in both patients (52).

Since SARS-CoV-2 can affect neuronal cells by both direct and indirect mechanisms, this can lead to various neurological manifestations also including anosmia and hypogeusia. Anosmia and hypogeusia are present both in mild/moderate cases and in severe cases of COVID-19 (45, 47, 49, 57, 60, 62, 64, 66–68, 118–122). As long-term COVID-19 symptoms, anosmia, and hypogeusia were evaluated in severe COVID-19 patients only in one protective study (67). The study evaluated 138 COVID-19 patients at 2 months of follow-up showing that 5.8% of patients had moderate to severe olfactory dysfunction, while 4.3% had a significant taste disorder (67). A greater number of studies evaluated olfactory and gustatory disfunctions in mild COVID-19 patients (45, 47, 49, 64, 66, 68, 118). Using a retrospective questionnaire Fjaeldstad evaluated olfactory and gustatory loss in 109 mild COVID-19 patients (49). At ~1 month after symptoms onset since the chemosensory loss, participants reported relatively low recovery and improvement rates. For participants with olfactory loss, only 44% were fully recovered, whereas 28% had not yet experienced any improvement of symptoms (49). After gustatory loss, 50% had fully recovered, whereas 20% had not yet experienced any improvement. At a longer follow-up of 2 months after symptoms onset, Otte et al. evaluating through a questionnaire 91 mild COVID-19 patients for olfactory function, showed that 45.1% of patients were hyposmic while 53.8% showed an olfactory performance within the normal range (118). In the same way, at 2 months of follow-up, Boscolo-Rizzo et al. evaluated prospectively 183 mildly symptomatic COVID-19 patients showing that 18.6% presented altered sense of smell or taste (45). Interestingly, Ugurlu et al. in a cohort of mild COVID-19 patients showed persistent olfactory dysfunction in 14.3% of patients up to 3 months after symptoms onset (66). At the same follow-up, a long-term anosmia was also reported in a case-report of a 40-year-old woman with a mild COVID-19 diagnosis (64). Differently, other studies analyzing mild and asymptomatic COVID-19 patients for smell and taste disturbance reported resolution of anosmia up to 1 month after diagnosis (47, 60). Comparable results were also reported at the same follow-up by Konstantinidis et al. evaluating mild/moderate COVID-19 patients (119). Finally, Paolo et al., analyzing 75 mild-to moderate COVID-19 patients through a questionnaire reported olfactory and dysgeusia recovery within an average of 17 days, also finding a significantly decrease in viral load (120).

Finally, other studies evaluating heterogenous populations of mild-to-severe COVID-19 patients further confirmed persistent loss of smell up to 6 months after symptom onset (57, 61, 62, 64, 121, 122). Moein et al. in a prospective study on 82 mild-to-severe COVID-19 patients showed smell loss in ~37% of patients at 2 months of follow-up (57). At the same follow-up, a prospective study on 138 patients and a retrospective study on 90 mild-to-severe patients showed persistent hyposmia in 5–8% of patients (57, 67). Lastly, Pilotto et al., by examining retrospectively 165 patients detected the presence of hyposmia in ~15% of patients at up to 6 months of follow-up (61).

#### Widespread Persistent Symptoms

Numerous research groups reported widespread persisting symptoms in COVID-19 patients for up to 6 months after SARS-CoV-2 infection (70, 75, 123–142). They also described practice on the management of post-acute COVID-19 and performed comprehensive analyses of health-related quality of life (70, 75, 123). Furthermore, numerous editorials, reviews, news articles, clinical updates, narrative interviews, and focus groups have been published to explore what it is like to live with long-term COVID-19, also trying to emphasize the putative pathophysiology, risk factors, and treatments (124–142). Two cohort studies on severe/critical COVID-19 patients reported persistent physiological impairment and decrease in quality of life in more than half of the patients at up to 6 months of follow up (77, 100). Taboada et al. showed that at 6 months of follow-up only 16% of patients were completely free of persistent symptoms (100). However, in a Multistate Health Care Systems Network, Tenforde et al. reported that among 270 interviews conducted on COVID-19 patients, also among persons with milder outpatient illness, 14–21 days after symptoms onset, the 35% of patients had not returned to their usual state of health (143). In this context, Pellaud et al., examining the outcomes of 196 consecutively mild-moderate COVID-19 patients, 1 month after onset of symptoms, showed that among the 60% of patients that returned home, 63% reported persistent symptoms (90). Two months after symptom onset, evaluating 150 mild/moderate COVID-19 patients, Carvalho-Schneider et al. showed that about 66% of patients presented at least one symptom (74). Similarly, evaluating the long-term COVID-19 symptoms in 233 mild COVID-19 patients Cirulli et al. highlighted that ~24% of patients had at least one symptom also after 3 months (76). These results were also confirmed by an online survey of doctors conducted by the *British Medical Association* (144). They reported that of 3,729 doctors who answered a question about patients' persistent symptoms after COVID-19, a third said that they had seen or treated patients with long-term COVID-19 symptoms (144). Davido et al. also reported that since mid-May they evaluated an average of 30 individuals per week for whom COVID-19 symptoms have not completely subsided, essentially young women (sex *ratio* 4:1) around 40 years old with no relevant medical history (145–147). Additionally, it was reported that female sex (mean age 47.22) is also a risk factor for poor health-related quality of life in Chinese COVID-19 patients (75). Also, Sudre et al. analyzing 4,182 incident cases of non-severe COVID-19 who logged their symptoms prospectively in the COVID-19 Symptom Study App showed that women aged 50–60 were at greatest risk of developing “long-COVID” (148). Patients described symptoms in every part of the body which were sometimes severe or fluctuating (149, 150). Paul Garner, a professor at Liverpool School of Tropical Medicine and Co-ordinating Editor of the Cochrane Infectious Diseases Group, wrote on the 95th day after symptoms onset in the *British Medical Journal Opinion* (151). He said “*I am unable to be out of bed for more than three hours at a stretch…I have ringing in the ears, intermittent brain fog, palpitations, and dramatic mood swings*” (151). Other people also described similar complaints in the same journal (152–154). The science journalist Linda Geddes also discussed data from the Irish Centre for Vascular Biology in Dublin that reported COVID-19 patients being discharged from hospital, only to return several weeks later not only with widespread symptoms but also with deep vein thrombosis or blood clots on the lungs (155).

The main widespread reported long-term symptoms in COVID-19 patients were chronic fatigue, dyspnea, shortness of breath, chest pains, headache, loss of smell/taste, muscle, and joint pain, followed by depression, anxiety, insomnia, and itchy body, heart palpitations, tachycardia, anorexia, tingling fingertips, and brain fog (69, 70, 72, 77, 84, 85, 87, 91, 97, 98, 101, 103, 123, 138, 145–147, 150, 156–159). However, it was reported that the number of widespread long-term symptoms were higher for COVID-19 patients who were initially more ill (77, 100). D'Cruz et al. and Taboada et al., analyzing prospectively two cohorts of 119 and 91 severe/critical COVID-19 patients, respectively, showed the presence of dyspnoea on exertion (57%), asthaenia (37%), myalgia (37%), and arthralgia (29%) up to 2 months after symptoms onset and a general decrease in quality of life (mobility, usual activities, self-care, pain/discomfort, anxiety/depression) in 67% of patients at up to 6 months of follow-up (77, 100). However, these widespread long-term symptoms were not only present in severe COVID-19 patients, but also in patients who had mild and moderate disease (72, 76, 80, 94, 98, 146, 159). Carvalho-Schneider et al., in a prospective study on 150 mild/moderate COVID-19 patients at 2 months of follow-up, highlighted dyspnea and asthenia, respectively, in 30 and 40% of patients (74, 98). Similar results were also obtained in a cross-sectional study on 451 mild COVID-19 patients (98). In addition to these symptoms in a prospective study by Cirulli et al. symptoms such as difficulty concentrating, fatigue, memory loss, confusion, headache, heart palpitations, chest pain, pain with deep breaths, dizziness, and tachycardia were detected at 3 months of follow up (76). Fatigue, dyspnea, and heart dysfunctions in mild COVID-19 patients were also reported in several case-reports (69, 72, 80) at up to 8 months of follow-up. In addition, a case-report on three women also reported telogen effluvium, temporary hair shedding, as a long-term COVID-19 symptom 3 months after getting the infection (94). Several studies analyzing all together mild-to-severe COVID-19 patients also confirmed these long-term widespread symptoms (73, 78–81, 83, 88, 89, 92, 93, 95, 99, 102–104, 160–162). In a large cohort of 355 mild-to-severe COVID-19 patients Mahmud et al. detected that about 46% of patients developed long-term symptoms at 1 month of follow-up and that post-COVID features were significantly higher among the female gender with fatigue as the main long-term symptom (88). Similarly, persistent fatigue was also reported in about 12% of patients by examining a cohort of 1,002 mild-to-severe COVID-19 patients (95). At a longer follow-up, Rosales-Castillo et al. and Townsend et al. confirmed persistence of fatigue as the main long-term symptom in a cohort of mild-to-severe COVID-19 patients (93, 102). Banda et al., analyzing 150 *tweets* from moderate-to-severe COVID-19 patients, reported that the 10 most commonly long-term symptoms after COVID-19 were chronic fatigue (62%), dyspnea (19%), tachycardia/palpitations (13%), chest pain (13%), sleep disorders (10%), cough (9%), headache (7%), and joint pain, fever, and unspecified pain by 6% each (160). This analysis also matches clinician-collected data reported by an Italian study (73). The study followed 143 hospitalized mild-to-severe patients who were discharged from the hospital after COVID-19 and that had two negative test results for SARS-CoV-2 (73). At an average of 2 months after initial onset of symptoms, “*only 12.6% were completely free of any COVID-19-related symptom, while 32% had 1 or 2 symptoms and 55% had 3 or more*” (73). Also, in this case the most common symptoms were chronic fatigue (53.1%), dyspnea (43.4%), joint pain (27.3%), and chest pain (21.7%) (73). Authors also observed that individuals who had an initial symptom of dyspnea were more likely to develop long-term symptoms (73). These results were also confirmed by a case-control study that examined 141 mild-to-moderate COVID-19 patients and 78 controls at 2 months of follow-up (79). At the same follow-up a retrospective study on 206 mild-to-moderate COVID-19 patients also detected chronic pain in ~40% of the patients (78). A particular cross-sectional study on 46 mild-to-severe COVID-19 patients also described an extremely low outlier *ratio* of total protein, albumin, and globulin at 2 months of follow-up, underlying a persistent abnormal liver function (104). At the same follow-up lymphopenia, elevated D-dimer, and C reactive protein were also detected and associated to persistent fatigue, dyspnea, and anosmia (87, 103). Fatigue and dyspnea were also the two most prevalent persistent symptoms 3 months after a SARS-CoV-2 infection in hospitalized and non-hospitalized patients (83, 92, 103, 161). Furthermore, at the same follow-up, Raman et al. also reported abnormalities in heart (26%), liver (10%), and kidneys (29%) (92). Dyspnea (42%), associated with chronic fatigue (55%), loss of memory (34%), concentration and sleep disorders (28 and 30.8%, respectively), was likewise reported in 120 COVID-19 patients (relatively non-severe) analyzed by questionnaire, 100 days after initial symptoms onset (162). In was also shown that these long-term symptoms persisted for up to 6 months, with fatigue or muscle weakness and sleep difficulties as the most common symptoms (81, 99). At 6 months, by examining 9,989 mild-to-severe COVID-19 patients, persistent neuropsychiatric, pulmonary, metabolic, and coagulopathic phenotypes were also reported (89).

Recent data reported several of these widespread long-term symptoms, i.e., fatigue, dyspnea, chest pains, muscle and joint pain, headache, insomnia, and palpitations, also in children and adolescent up to 6–8 months of follow-up (71, 86, 96). At 6 months of follow-up high blood pressure levels and persistence of a prehypertension were also detected in ~13% of mild COVID-19 children (82). Examining a larger cohort of children, it was also described that ~53–70% of these patients had at least one symptom 100 days or more after COVID-19 diagnosis (71, 82, 96, 163). Given these emerging data, recently, Hertting et al. in an editorial on *Acta Paediatrica* underlined the need to have more research and studies on the long-term effects of COVID-19 in children and adolescents (164).

## Discussion

Although we are aware that there are no long-term data on large numbers of COVID-19 patients with persistent symptoms and with comparison groups, and that an analysis in a field as engaging as COVID-19 can never be updated, this review allowed us to outline that a noteworthy number of patients present long-lasting *sequelae*, up to 6 months, in the post-COVID time. These long-term symptoms are not only present in severe COVID-19, but also in mild and moderate patients. In addition, recent preliminary data also underlined the presence of long-term COVID-19 symptoms on children and adolescents. Some clinical studies and survey questionnaires also highlighted a potential high-risk factor for long-term COVID-19 in the female gender; women's risk of developing long-term COVID-19 seems to be double that of men among patients aged between 40 and 50. After the age of 60 the risk level of long-term COVID between male and female should become similar. This pattern appears to be like that of autoimmune diseases that are more common in female through menopause to become similar between male and female after age 60 (165). Thus, it is possible that these gender differences, as well as other aspects of the disease, may be due to a different immune system response during and after COVID-19. However, currently, it is not yet clear whether this data reflects the population of people with long-term COVID-19 and which is the full spectrum of the duration and severity of long-term symptoms in these patients.

What emerges from this review is that the most common reported symptoms after COVID-19 are abnormal lung functions prevalently with persistent dyspnea, general neurological decay, smell and taste disturbances, and chronic fatigue. Other common symptoms include joint pain and chest pain. These symptoms may linger or recur for weeks or months following initial recovery. In detail, for patients with mild-to-moderate COVID-19 the more common long-term symptoms are chronic fatigue, anosmia/ageusia, dyspnea, but also difficulty in concentration, memory loss, and confusion. These symptoms seem to be present in a higher percentage of patients who were initially more ill. In critical-to-severe COVID-19 patients' supplementary long-term symptoms are lung fibrotic-like changes up to 6 months after infection and a high reduction in diffusing capacity of the lung for D_LCO_ that frequently required oxygen uses also after hospital discharge. Likewise, the general cognitive decay, despite also being present in mild-to-moderate COVID-19 patients, also appears to be more closely related to critical-to-severe forms of COVID-19. Considering the whole overview of widespread long-term symptoms reported in this review the one undeniably most prevalent in mild-to-critical COVID-19 patients is chronic fatigue. This is in line with past research that highlighted high levels of post-infectious fatigue for survivors of epidemics such as SARS and Ebolavirus (166, 167). Moreover, fatigue has been related with infections, such as mononucleosis, that occur outside of an epidemic or pandemic scale (167). Currently, it is not clear why chronic fatigue and the other long-term complications persist in some COVID-19 patients. However, most researchers and clinicians agree that the long-term COVID-19 symptoms are associated with the coronavirus' ability to trigger a massive inflammatory response. Thus, it will be mandatory to analyze cytokine networks in patients who recover from COVID-19 to evaluate whether the “cytokine storm” present during the disease persists and contributes to these long-term complications.

The main strength of this study is that it highlights multiple long-term symptoms which may hinder return to pre-COVID-19 infection functional status. However, despite this finding a weakness of our review was that while some studies included in this review focused on a single population of infected COVID-19 patients, i.e., mild/moderate and severe/critical, numerous studies included heterogeneous populations, from mild to critical, not taking into account disease severity as well as preexisting co-morbidities, treatment regimens, mean ages, gender, and other aspects. This bias can lead to alterations in the data evaluation and analysis, which potentially affect the results. Data from prospective designs, developed by evaluating homogeneous populations of COVID-19 patients able to consider their characteristics prior to and during infection, might provide new and detailed information into predisposing factors that lead to long-term COVID-19 symptoms. Another bias that should be considered is that despite the fact that in some studies the long-term COVID-19 *sequelae* were evaluated through clinical visits and/or specific instrumental analyzes, many others have used self-administered questionnaires and scores, telephone/online interview, and phone applications. This is because, to date, assessing the patient in the hospital is difficult due to the entry restrictions into the COVID-19 departments. On the other hand, checking and evaluating them at home presents almost insurmountable logistical problems during an emergency health situation like the one we are facing. However, this type of self-assessment highlights bias in the detection of symptoms as patients may have psychological and emotional involvement due to the disease itself.

At this stage, a detailed analysis and understanding of all the aspects associated with long-term COVID-19 are mandatory to mitigate against the potential persistent symptoms identified in the current review. Future studies should assess: (1) the full range of disorders associated with COVID-19 and their long-term manifestations; (2) the underlying associations between viral spread, associated pro-inflammatory changes, and long-term disease pathogenesis; (3) the duration and extent of long-term symptoms in relation to the resolution of the disease; (4) the association between disease severity and long-term dysfunctions; (5) the effect of specific antiviral therapies and/or interventions on long-term symptoms; (6) why symptoms persist or recur; and (7) the potential late effects of COVID-19 on children/adolescents. Another important point that should be assessed is SARS-CoV-2 levels (detection, load) in patients and how this relates to long-term symptoms. To date, it is not clear whether the initial viral load, *per se*, may meaningfully impact long-term symptoms, particularly in mild-to-moderate COVID-19 patients. Information relating to SARS-CoV-2 detection and viral load at different time points of infection will help the clinical interpretation of long-term symptoms of COVID-19. Similarly, there is a need for further studies to provide robust data on the association between viral shedding and long-term COVID-19. Despite has reports that the median duration of viral shedding goes from 12-to-20 days, there is evidence that ongoing viral shedding in SARS-CoV-2 may be prolonged in the feces compared to respiratory secretions (168–170). The persistent fragments of viral genes, though not infectious, may still be triggering a violent immune overreaction that could explain the symptoms persistence in COVID-19-free patients. Alternatively, even if the virus is cleared, the immune system could continue to be overactive or perturbed, analogous to the long-term debilitation after glandular fever (165). A greater understanding of these last points could improve the knowledge not only of the causes of long-term symptoms but also on the immune system involvement and on transmission risk.

## Data Availability Statement

The original contributions presented in the study are included in the article/Supplementary Material, further inquiries can be directed to the corresponding author/s.

## Author Contributions

FS and MF designed the review. FS and FV performed the literature search and collected and assembled the data. FS, FV, and MF analyzed the obtained articles. FS, FV, ML, and MF wrote the paper. MF, LM, and ML revised the manuscript critically. All authors have read and agreed to the published version of the manuscript.

## Conflict of Interest

The authors declare that the research was conducted in the absence of any commercial or financial relationships that could be construed as a potential conflict of interest.
