# Supplementary material for: Post-COVID-19 Syndrome: The Persistent Symptoms at the Post-viral Stage of the Disease. A Systematic Review of the Current Data
Source: Front Med (Lausanne). 2021 May 4;8:653516. doi: 10.3389/fmed.2021.653516 (PMC8129035; doi:10.3389/fmed.2021.653516)
Supplement: Supplementary file 1 [file Table_1.pdf]

Table: Quality Assessment Tool for Observational Cohort and Cross-Sectional Studies.

Reference	Criteria													
	**1**	**2**	**3**	**4**	**5**	**6**	**7**	**8**	**9**	**10**	**11**	**12**	**13**	**14**
Arnold et al. [88]								NA						NR
Bellan et al. [39]								NA				CD		
Boscolo-Rizzo et al. [77]								NA					NR	
Buonsenso et al. [164]								NA						NR
Carfì et al. [151]								NA						
Carvalho-Schneider et al. [113]								NA						NR
Caronna et al. [63]								NA					NR	NR
Chen et al. [75]								NA						
Chun et al. [32]								NA						NR
Cirulli et al. [114]								NA	NR		NR			NR
Daher et al. [33]								NA						NR
D'Cruz et al. [109]								NA						NR
Ding et al. [47]								NA						NR
Erçalık et al. [153]								NA						
Frija-Masson et al. [37] *								NA						NR
Fjaeldstad et al. [75]								NA					NR	
Galal et al. [62]								NA					NR	NR
Gallus et al. [56]								NA						
Hall et al. [30]*														CD
Han et al. [31]								NA						NR
Hu et al. [48]				NR				NA					NR	NR
Huang et al. [160]								NA						
Isoldi et al. [166]								NA						NR
Iqbal et al. [157]								NA						
Jacobs et al. [132]								NA						
Khalaf et al. [133]								NA	CD		CD		NR	CD
Latronico et al. [29]								NA	CD		CD			NR
Liang et al. [24]								NA						
Liao et al. [49]								NA						CD

Lu et al. [71]								NA						NR
Mo et al. [50]								NA					NR	NR
Mandal et al. [134]								NA	CD		CD			CD
Mahmud et al. [146]								NA						
Martin et al. [162]			NR					NA	CD		CD		NR	CD
Mendez et al. [66]								NA						
Moein et al. [84]								NA						NR
Moreno-Perez et al. [28]								NA						CD
Panda et al. [80]								NA						
Pellaud et al. [112]								NA						NR
Petersen et al. [135]								NA						CD
Pilotto et al. [65]								NA						CD
Pritza et al. [85]								NA						
Raman et al. [158]								NA						
Ramakrishnan et al. [40]								NA						
Rosales-Castillo et al. [148]*			NR					NA	NR		NR		NR	NR
Saiful Islam et al. [147]								NA						
Shah et al. [27]								NA	CD		CD			CD
Smane et al. [165]								NA	NR		NR			
Sykes et al. [161]								NA						
Sonnweber et al. [46]				NR				NA					NR	NR
Stavem et al. [137]								NA						
Tabatabaei et al. [25]								NA	NR		NR		NR	CD
Taboada et al. [110]								NA	CD		CD			NR
Townsend et al. [138]								NA					NR	
Townsend et al. [149]								NA						CD
Trinkmann et al. [42]*			NR					NA					NR	NR
Truffaut et al. [34]*								NA					CD	
Ugurlu et al. [78]								NA						NR
Van den Borst et al. [26]								NA	NR		NR		NR	CD
Van Gassel et al. [36]*			NR					NA					NR	NR
Vaira et al. [72]								NA						
Varghese et al. [155]			CD					NA	CD		CD		NR	CD

Weerahandi et al. [22]	Yes	Yes	Yes	Yes	No	Yes	Yes	NA	Yes	Yes	Yes	No	Yes	Yes
Ya-Wen An et al. [154]	Yes	Yes	Yes	Yes	No	Yes	Yes	NA	No	Yes	No	No	Yes	NR
Yan et al. [74]	Yes	Yes	CD	Yes	No	Yes	Yes	NA	CD	No	CD	No	CD	NR
Zhao et al. [38]	Yes	Yes	Yes	No	No	Yes	Yes	NA	Yes	Yes	Yes	No	Yes	NR

*Cohort studies and cross-sectional studies published as Letters to Editor or as brief communication

1. Was the research question or objective in this paper clearly stated? **2.** Was the study population clearly specified and defined? **3.** Was the participation rate of eligible persons at least 50%? **4.** Were all the subjects selected or recruited from the same or similar populations (including the same time period)? Were inclusion and exclusion criteria for being in the study prespecified and applied uniformly to all participants? **5.** Was a sample size justification, power description, or variance and effect estimates provided? **6.** For the analyses in this paper, were the exposure(s) of interest measured prior to the outcome(s) being measured? **7.** Was the timeframe sufficient so that one could reasonably expect to see an association between exposure and outcome if it existed? **8.** For exposures that can vary in amount or level, did the study examine different levels of the exposure as related to the outcome (e.g., categories of exposure, or exposure measured as continuous variable)? **9.** Were the exposure measures (independent variables) clearly defined, valid, reliable, and implemented consistently across all study participants? **10.** Was the exposure(s) assessed more than once over time? **11.** Were the outcome measures (dependent variables) clearly defined, valid, reliable, and implemented consistently across all study participants? **12.** Were the outcome assessors blinded to the exposure status of participants? **13.** Was loss to follow-up after baseline 20% or less? **14.** Were key potential confounding variables measured and adjusted statistically for their impact on the relationship between exposure(s) and outcome(s)?

Yes **No**

CD, cannot determine; **NA,** not applicable; **NR,** not reported

Table: Quality Assessment Tool for Case-Control Studies.

Reference	Criteria											
	**1**	**2**	**3**	**4**	**5**	**6**	**7**	**8**	**9**	**10**	**11**	**12**
D’Ascanio et al. [79]	Yes	Yes	Yes	Yes	Yes	Yes	Yes	Yes	Yes	Yes	NR	Yes
Galvan-Tejada et al. [152]	Yes	Yes	CD	Yes	Yes	Yes	NR	Yes	Yes	NR	NR	Yes

1. Was the research question or objective in this paper clearly stated and appropriate? **2.** Was the study population clearly specified and defined? **3.** Did the authors include a sample size justification? **4.** Were controls selected or recruited from the same or similar population that gave rise to the cases (including the same timeframe)? **5.** Were the definitions, inclusion and exclusion criteria, algorithms or processes used to identify or select cases and controls valid, reliable, and implemented consistently across all study participants? **6.** Were the cases clearly defined and differentiated from controls? **7.** If less than 100 percent of eligible cases and/or controls were selected for the study, were the cases and/or controls randomly selected from those eligible? **8.** Was there use of concurrent controls? **9.** Were the investigators able to confirm that the exposure/risk occurred prior to the

development of the condition or event that defined a participant as a case? **10.** Were the measures of exposure/risk clearly defined, valid, reliable, and implemented consistently (including the same time period) across all study participants? **11.** Were the assessors of exposure/risk blinded to the case or control status of participants? **12.** Were key potential confounding variables measured and adjusted statistically in the analyses? If matching was used, did the investigators account for matching during study analysis?

Yes **No**

CD, cannot determine; **NA,** not applicable; **NR,** not reported

Table: Quality Assessment of case series and case reports.

Reference	Selection	Ascertainment		Causality				Reporting
	1	2	3	4	5	6	7	8
Abdallah et al. [141]						NA		
Buselli et al. [140]						NA		
Dani et al. [67]						NA		
Guedj et al. [53]						NA		
Hellmuth et al. [57]						NA		
Heiss et al. [23]						NA		
Hosseini et al. [145]						NA		
Lim et al. [58]						NA		
Ludvigsson et al. [163]						NA		
Manckoundia et al. [41]						NA		
Negrini et al. [54]						NA		
Novak et al. [68]						NA		
Raahimi et al. [69]						NA		
Sampaio Rocha-Filho et al. [73]						NA		
Saeed et al. [144]						NA		
Tobechukwu et al. [59]						NA		
Yao et al. [21]*						NA		
Zhu et al. [35]						NA		

*Case-reports published as Letters to Editor

1. Does the patient(s) represent(s) the whole experience of the investigator (centre) or is the selection method unclear to the extent that other patients with similar presentation may not have been reported? **2.** Was the exposure adequately ascertained? **3.** Was the outcome adequately ascertained? **4.** Were other alternative causes that may explain the observation ruled out? **5.** Was there a challenge/rechallenge phenomenon? **6.** Was there a dose–response effect? **7.** Was follow-up long enough for outcomes to occur? **8.** Is the case(s) described with sufficient details.

Yes **No**

CD, cannot determine; **NA,** not applicable; **NR,** not reported

Table: Quality Assessment of Systematic Reviews and Meta-Analyses.

**Reference**	**Criteria**							
	**1**	**2**	**3**	**4**	**5**	**6**	**7**	**8**
Amenta et al. [91]	Yes	No	No	No	No	No	No	NA
Bougakov et al. [52]	Yes	No	No	No	No	Yes	No	NA
Candan et al. [55]	Yes	No	No	No	No	Yes	No	NA
Fischer et al. [61]	Yes	Yes	Yes	No	No	Yes	No	NA
Higgins et al. [91]	Yes	No	No	No	No	Yes	No	NA
Lopez-Leon [98]	Yes	Yes	Yes	Yes	Yes	Yes	No	NA
Nikhra et al. [101]	Yes	No	No	No	No	Yes	No	NA
Shaw et al. [45]	Yes	No	No	No	No	Yes	No	NA
Yong et al. [107]	Yes	No	No	No	No	Yes	No	NA
Zapatero et al. [108]	Yes	Yes	No	No	No	Yes	No	NA

1. Is the review based on a focused question that is adequately formulated and described? **2**. Were eligibility criteria for included and excluded studies predefined and specified? **3**. Did the literature search strategy use a comprehensive, systematic approach? **4**. Were titles, abstracts, and full-text articles dually and independently reviewed for inclusion and exclusion to minimize bias? **5**. Was the quality of each included study rated independently by two or more reviewers using a standard method to appraise its internal validity? **6**. Were the included studies listed along with important characteristics and results of each study? **7**. Was publication bias assessed? **8**. Was heterogeneity assessed? (This question applies only to meta-analyses).

Yes **No**

CD, cannot determine; **NA,** not applicable; **NR,** not reported
